# Sequentially based analysis versus image based analysis of Intima Media Thickness in common carotid arteries studies - Do major IMT studies underestimate the true relations for cardio- and cerebrovascular risk?

**DOI:** 10.1186/1476-7120-6-32

**Published:** 2008-06-20

**Authors:** M Sandrock, J Hansel, J Schulze, D Schmitz, A Niess, H Burkhardt, A Schmidt-Trucksaess

**Affiliations:** 1 Freiburg University Hospital, Centre for Internal Medicine, Department for Rehabilitative and Preventative Sports Medicine, D-79106 Freiburg, Germany; 2 Technical University Munich, Department for Sports Medicine, D-80106 Munich, Germany; 3 Medical Clinic, Department of Sports Medicine, University of Tuebingen, D-72074 Tuebingen, Germany; 4 Freiburg Informatik University, Abt. für Bildverarbeitung, D-79106 Freiburg, Germany

## Abstract

**Background:**

Image-based B-mode ultrasound has gained popularity in major studies as a non-invasive method of measuring cardio- and cerebrovascular risk factors. However, none of the major studies appears to have paid sufficient attention to the variation in end diastolic wall process. By using sequentially based analyses (SBA) of Intima-Media Thickness (IMT), the general purpose of this study was to show that the current image based (ECG tracked) analysis (IBA) has some major variations and might underestimate the true relations for cardiovascular events and stroke for IMT measurement.

**Method:**

The study group consisted of 2500 healthy male subjects aged between 35 to 55 years. 4 sequences (300 images) were analyzed per subject. 750,000 images were analysed throughout the course of this study.

**Results:**

IBA showed significantly lower mean, maximal, and minimal values for IMT in CCA than for SBA. The correlation analysis between IBA and SBA with the cardio- and cerebrovascular risk factors showed a higher correlation of SBA for all risk factors. The Pearson coefficient was 0.81, p < 0.01, for SBA versus Framingham CHD risk level (FCRL) and 0.49, p = 0.01, for IBA versus FCRL.

**Conclusion:**

IBA did not measure the true maximal values of the IMT in this study. Together with the correlation analysis, this indicates that IBA might underestimate the true relations for IMT and risk factors.

## Introduction

Over the last few years, numerous studies have linked carotid Intima Media Thickness (IMT) and IMT progression with prevalent symptomatic coronary artery disease (CAD) [1-5] and cerebrovascular disease [1,4-7], as well as with cerebral white matter lesions[8,9]. Increased IMT as identified by B-mode ultrasound of the extra cranial carotid arteries is a predictor of incident coronary events [1,10,11], stroke [10] and all-cause mortality [12].

O'Leary and colleagues [10] reported that the risk of incident stroke or myocardial infarction adjusted for age and sex increased among Cardiovascular Health Study participants. In the Atherosclerosis Risk in Communities study, the addition of IMT to a basic model including traditional risk factors increased the area under the receiver-operator curve for stroke, but this was only statistically significant when combined with markers of peripheral vascular disease [13]. In a head-to-head comparison, CT-identified coronary artery calcium (CAC) discriminated CAD patients from controls better than IMT [14].

It seems logical to develop additional ultrasound methods to measure the risk of cardio- and cerebrovascular events and stroke more precisely.

The carotid artery seems to be a useful window for cardio- and cerebrovascular risk, with ultrasound able to provide unique information on morphological, hemodynamic and elastic properties[15]. The majority of clinical wall motion studies have derived elasticity parameters from measurements of the wall motion at a single location in vessels. Vessels have included the common carotid artery [16,17], femoral artery [16], brachial artery [17,18], cerebral arteries [19] and aorta [20,21].

Various ultrasound techniques have been used to detect and track vessel wall motion – manual as well as computer based measurements. Computational techniques have mainly been based on analysis of the B-mode greyscale images [18], M-mode [17] and analyses of the raw RF ultrasound data.

All methods – manual and automatic – have one main part in common; all methods use an end diastolic ECG tracked image [17]. Is this truly the maximal value during the systolic and diastolic time period?

In heart rate regulation, baroreflex plays a very important role, and is commonly related to carotid baroreceptor function. Systemic changes of the blood vessel wall and heart contraction may affect carotid artery pulse wave phase characteristics: carotid artery dilates more or less, decreases or increases the impulsation from baroreceptors. Because of that, heart rate variability changes in the baroreflex band of the frequency spectrum. Murgo et al [22,23] divide arterial systolic pressure pulsation into three classes, based on the timing and prominence of the secondary wave caused by reflections. Each class has a different restoring force for the IMT. Due to these variations in systolic movement, the point in time of the end diastolic maximal expanse of the IM layer differs from cycle to cycle. Based on this concept and knowledge about the variety of HR variation, a measurement of end diastolic IMT images seems to be very complicated due to the class of arterial pulse pressure. We think that the computer can solve this problem by calculating each image during the end diastolic period. The maximal value of this sequence should be independent from heart cycle regulations.

Most of the major studies about IMT in major journals[10,24] have not dealt adequately with this fundamental problem of IMT measurement. Due to this, they have more measurement error (primary error) and thus the reported relations are likely an underestimation of the true underlying relation of IMT compared to cardio- and cerebrovascular risk factors.

The aim of this study was to evaluate the correlation of image based IMT measurement to sequentially based analysis and cardio- and cerebrovascular risk factors.

## Materials and methods

### Study population

The study group consists of 2500 healthy male subjects (HS) aged between 35 to 55 years (age: 46.1 ± 5.6 years). All subjects underwent a detailed interview, completed a questionnaire (26) to evaluate their physical activity and food habits, a clinical examination, an ultrasound examination of the carotid tree, and a blood test for the lipid profile.

All subjects were free of cardiovascular disease as determined by medical history. The characteristics of the study population are given in Table 1.

**Table 1:** Characteristics of the study subjects (Anthropometry)

Subjects	Subjects
Number of subjects	2500
Age (years)	46.13 ± 5.6
Weight (kg)	73.7 ± 14.9
Height (cm)	174.6 ± 8.5
Body Mass Index (kg/m^2^)	23.1 ± 4.5
Systolic (mmHg)	117.3 ± 14.7
Diastolic (mmHg)	82.9 ± 8.8
Cholesterol (mmol/l)	219.8 ± 40.1
Triglyceride (mmol/l)	172.6 ± 60.4
LDL (mg/dl)	127.4 ± 31.2
HDL (mg/dl)	59.1 ± 17.7
LDL:HDL (ratio)	2.3 ± 0.9
Smokers	688
Pack years	6.3 ± 3.2
Heart frequency rest (beats/min)	67.4 ± 13.3
Framingham CDH risk level	2.3 ± 1.7

Characteristics of the study subjects, LDL = low density protein, HDL = high density protein.

Subjects were advised to avoid caffeine intake for 8 h before the study and to maintain their usual lifestyle (diet, alcohol intake and exercise level) between studies. None of the subjects took any medications.

The research was carried out in accordance with the Declaration of Helsinki (1989) of the World Medical Association, and has been approved by the Freiburg University Research and Ethics Committee.

### Ultrasound Examination

After at least 15 minutes rest in supine position, each patient was scanned by six experienced ultrasonographic observers.

We used a Toshiba SSA-380A, Japan, ultrasound scanner with high resolution 10 MHz transducer and an aperture of 32 mm. The carotid tree was scanned the transducer positioned longitudinal to the CCA. The lumen was maximised and the position changed until the typical double lines of the intima and adventitia boundaries of the arterial wall could be seen. Both, near and far, boundaries could be seen in the scan.

**Figure 1:**
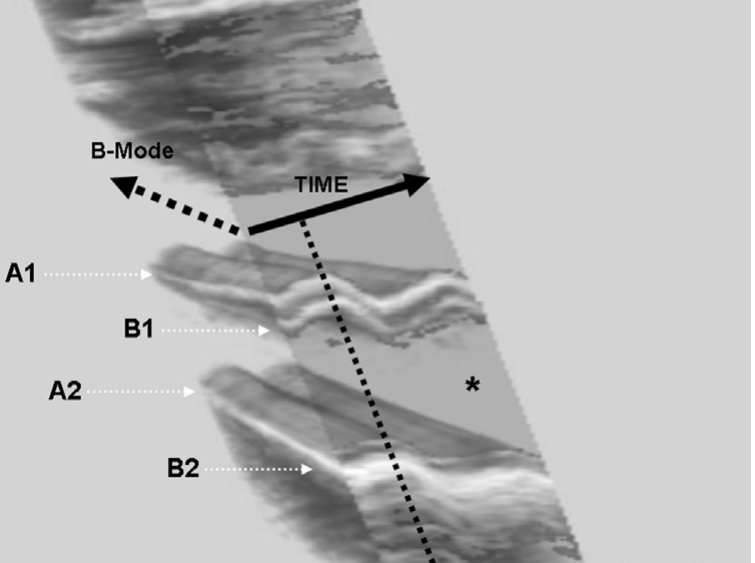
**Ultrasound sequence in 3D model**. The dotted arrow (z-axis) shows the B-mode image of ultrasound. The black arrow (x-axis) represents the time interval. The black dotted line represents the R-wave in the ECG. The marked star is positioned in the lumen of the artery. The wave motion movement of the artery can be seen clearly on the x-axis. The white arrows point to the contours of the intima media. A1 points to the near Media/Adventitia layer. B1 points to the near Intima/Media layer. A2 points to the near Media/Adventitia layer. B2 points to the near Intima/Media layer.

We stored one sequence for each subject for the lateral and for antero-lateral view for left and right CCA (4 sequences per subject) with the simultaneously recorded ECG at the top of the R-wave on digital disk (Sony DKR-700, Japan) (Figure 1).

Each sequence contains a time period of 3 seconds including at least 2 heart cycles. Each sequence was stored with 25 Hertz, which included 75 images per sequence. Additionally, all examinations were stored on videotape in order assist in the interpretation, if necessary.

After all we used 300 images per subject for the analysis (left and right artery, for every artery a lateral and for antero-lateral view, every sequence contains 75 images, 2 × 2 × 75 = 300). In the course of this study, 750,000 (2 × 2 × 75 × 2500 subjects) images for the sequence and 10,000 (2 × 2 × 2500 subjects) single images were analysed.

### Analysis of the images

All measurements and analyses were done by one experienced reader blinded to all clinical information. The reader visually controlled stored sequences of each subject.

### Image based analysis

For image based analysis (IBA), the reader chose the image from the digital videotape of the patient during the ultrasound examination with the R-wave in the ECG signal. This is the standardized procedure actually recommended by the recent consensus papers for IMT measurement.

This image was analysed for Image based Analyses of IMT measurement (Figure 1, dotted line). To understand this study, it is very important to emphasise that the image used for Image Based Analysis was part of the sequence later used for sequence analysis.

### Sequentially based analysis

**Figure 2:**
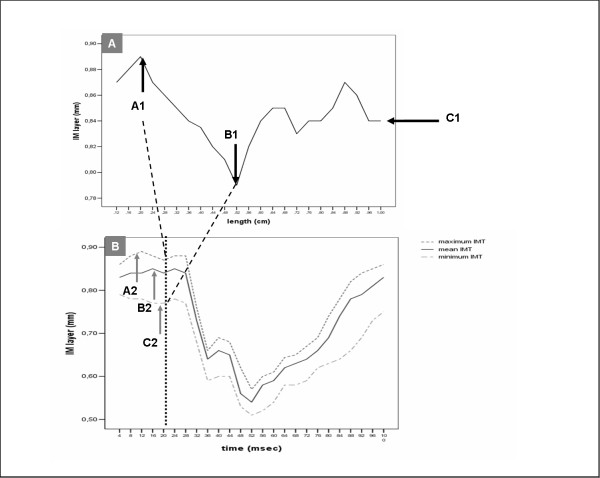
**Part A represents the image based analysis of an Intima Media layer analysis at the R-wave of the ECG (Image0).** The line represents the IMT value over the length of 1 cm proximal to the CCA opening to the Bulb in mm. A1 points to the Maximum value (0.87 mm), B1 to the Minimum (0.79 mm) and C1 to the Mean value of the IMT image based analysis. C1 marks the result for an image based mean IMT value (0.84 mm here). Part B represents the sequential based analysis of the sequence containing the image analysed in part A (black dotted line = Image0). The maximum (A2), minimum (B2) and mean values (C2) over the sequence are shown. It clearly shows that the value of A1 (0.87 mm) is less than A2 (0.90 mm).

The first sequentially based analysis was presented by Selzer et al., in 2001[25]. Based on this idea we used the same digital videotape sequence as described above (Figure 2) for the sequential based analysis (SBA). The same image chosen for the image based analysis was used as the starting point for the sequence analysis. The sequence array contains 75 images (SeqImage0–74). Because of this procedure, we reduced the variability in the measurement procedure due to rescanning, reselecting images and remeasuring processes to nearly zero.

For analysis of the IM layer we used a length of 1 cm from the beginning of the bulb backwards into the CCA like recommended by the recent consensus papers for IMT measurement [26].

We measured IMT at the far wall of the distal CCA and not at the near wall, because the detecting and trailing edge of the IM layers in the near wall lead to problems in the interpretation of the interfaces [27].

Results from left and right carotid artery were averaged.

### Parameters

#### Image based analysis

Mean Intima-Media Thickness (mIMT)

mIMT is defined as the mean value of the total IM layer in a chosen segment.

Maximal Intima-Media Thickness (maxIMT)

maxIMT is defined as the maximal value of the total IM layer in a chosen segment.

Minimal Intima-Media Thickness (minIMT)

minIMT is defined as the maximal value of the minimal total IM layer in a chosen segment.

#### Sequence based analysis

Maximal Mean Intima-Media Thickness (MmIMT)

MmIMT is defined as the maximal mean value of the total IM layer in a chosen sequence and segment.

Maximal Maximal Intima-Media Thickness (MmaxIMT)

maxIMT is defined as the maximal maximal value of the total IM layer in a chosen sequence and segment.

Minimal Intima-Media Thickness (MminIMT)

MminIMT is defined as the maximal minimal value of the total IM layer in a chosen sequence and segment.

### Assessment of Individual Coronary Heart Disease (CHD) Risk Level

For all subjects, the individual CHD risk level was estimated based on the Framingham CHD score sheets presented by Wilson et al. [28]. This scoring system was applied to subjects with an age ranging from 30 to 79 years. The details of the algorithm for determining the risk score are described in the original report. The higher the value of the score, the higher is the risk for a CHD.

### Statistics

All data were calculated using the software program SPSS (Software package for the social sciences) for Windows, Version 12. The arithmetic mean and the standard deviations (SD) were used for descriptive statistics.

Pearson's correlation coefficient was used to measure the strength of the linear relationship between IMT and WI (0.80–1.00: strong association, 0.60–0.79: strong-moderate association, 0.40–0.59: weak-moderate association)

A Bland & Altman analysis was performed to describe reproducibility of the method. The agreement for the reproducibility of WI was accepted if the upper and lower boundaries of the 95% confidence interval for WI covered zero.

## Results

### Characteristics of subjects

A total of 2500 subjects were examined in the study, including a total number of 750,000 images. Anthropometric data, resting heart rate, and blood pressure for both groups are shown in Table 1.

### Characteristics of IMT measurement

In the ultrasound analysis, IMT image based analysis showed *significantly lower *mean (IBA mean: 0.63 ± 0.18, SBA mean: 0.67 ± 0.19, p < 0.01), maximal (IBA max: 0.77 ± 0.19, SBA max: 0.83 ± 0.18, p < 0.01) and minimal (IBA min: 0.48 ± 0.15, SBA min: 0.51 ± 0.15, p < 0.01) values for the left, right, and combined CCA than for sequential based analysis (Table 2).

**Table 2:** Characteristics of the study subjects (Intima-media Thickness)

Parameters	Image Analysis	Sequential Analysis
IMT mean right CCA	0.64 ± 0.19	0.68 ± 0.20
IMT max right CCA	0.79 ± 0.19	0.84 ± 0.20
IMT min right CCA	0.49 ± 0.17	0.50 ± 0.16
IMT mean left CCA	0.63 ± 0.20	0.67 ± 0.20
IMT max left CCA	0.76 ± 0.21	0.83 ± 0.21
IMT min left CCA	0.47 ± 0.16	0.51 ± 0.18
IMT mean (left + right CCA)	0.63 ± 0.18	0.67 ± 0.19
IMT max (left + right CCA)	0.77 ± 0.19	0.83 ± 0.18
IMT min (left + right CCA)	0.48 ± 0.15	0.51 ± 0.15

Characteristics of the study subjects, CCA = common carotid artery. Image analysis was always performed during the first image with the first R-wave in the ECG. Sequential analysis was performed at the maximal value during the heart cycle.

### Bland-Altman analysis

The Bland-Altman analysis revealed a difference of 0.04 mm for maximal IMT common carotid artery and a correlation coefficient of 0.86 with systematically lower values for imaged based analysis than for sequential tracing in mean and minimum like the maximum IMT in left, right, and common carotid artery (Table 3).

**Table 3:** Sequential vs. image Bland-Altman

	Sequential vs. image Bland-Altman			
	Mean	Lower CI 95%	Higher CI 95%	r

Maximum right CCA	0.04	0.02	0.05	0.76
Mean right CCA	0.05	0.02	0.06	0.74
Minimum right CCA	0.02	0.01	0.03	0.76
Maximum left CCA	0.04	0.02	0.05	0.75
Mean left CCA	0.06	0.03	0.08	0.75
Minimum left CCA	0.04	0.03	0.05	0.76
Maximum left+ right CCA	0.04	0.01	0.05	0.76
Mean left+ right CCA	0.05	0.03	0.06	0.75
Minimum left+ right CCA	0.02	0.01	0.03	0.76

Values are presented for the mean and maximum (max) IMT and correlation coefficient of the common carotid artery (CCA) and the carotid bulb of the Bland-Altman analysis.

### Correlation analysis

**Figure 3:**
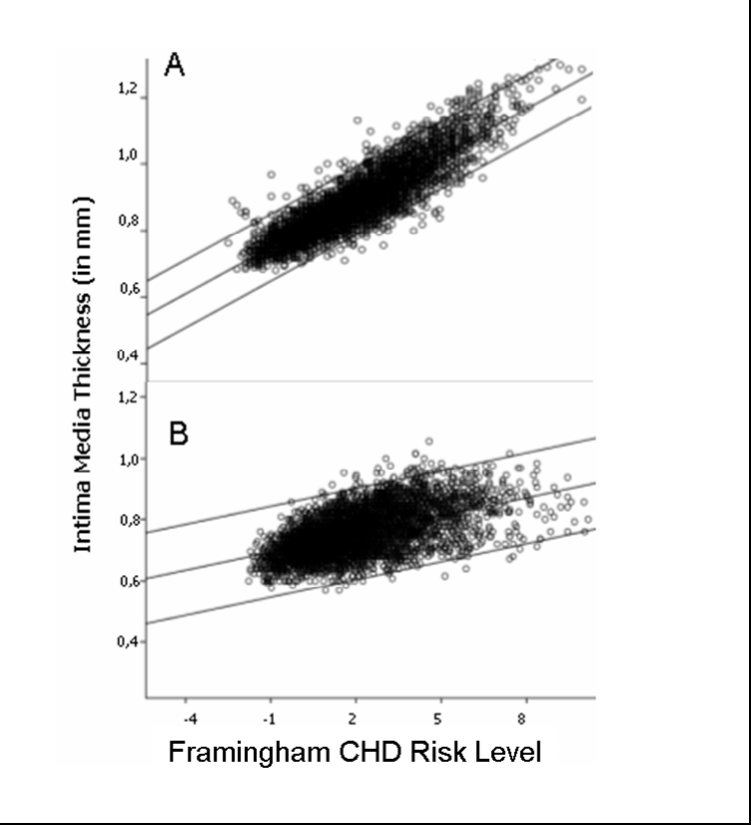
**Scatter plot for Intima Media Thickness versus the Frammingham CHD Risk Level**. Part A is for the sequential based analysis, B for the image based analysis. The Correlation line (middle black line) is given with the 95% confident interval (upper and lower black line).

The correlation analysis between IBA and SBA with the cardio- and cerebrovascular risk factors showed a higher correlation of SBA for all risk factors. The Pearson coefficient was 0.81, p < 0.01 for SBA versus Framingham, and 0.49, p = 0.01 for IBA versus Framingham. The same results were found for the other risk factors as well (Figure 3, Table 4).

**Table 4:** Correlation of image based IMT and sequential IMT

	Image based IMT		Sequential based IMT	
ECG position	Pearson	Sig.	Pearson	Sig.

Framingham CHD risk level	0.49	0.01	0.81	<0.01
HDL (mg/dl)	0.43	0.02	0.74	<0.01
LDL (mg/dl)	0.43	0.02	0.73	<0.01
Cholesterol (mmol/l)	0.37	0.03	0.57	<0.01
RR systolic (mmHg)	0.45	0.02	0.68	<0.01
RR diastolic (mmHg)	0.45	0.02	0.67	<0.01
LDL:HDL (ratio)	0.47	0.01	0.71	<0.01
Age (years)	0.46	0.01	0.71	<0.01
Pack years	0.37	0.03	0.65	<0.01

Correlation of image based IMT and sequential IMT compared to the risk factors of the subjects. Pearson = Pearson coefficient.

## Discussion

The main findings of this study are:

1) Sequential analysis could detect higher values in vivo situation of the maximal IM layers than image based IMT measurement (see *Characteristics *and Bland-Altman analysis).

2) Sequential analysis was significantly different from image based analysis

3) The results of correlation analyses may lead to the assumption that current IMT studies on image based analysis might underestimate the true relations of individual risk for atherosclerosis (see Correlation analysis).

First it is important to emphasize that this study used the same computer based detecting algorithm for the image based analysis as for the sequential based analysis. Using the same algorithm (25) reduces the errors of detecting the correct layers to a minimum.

The image used for the image analysis (IBA) was part of the sequence used for the sequential analysis. This is important to emphasize. Using this procedure – we used the same detection algorithm, the same reader, and the same video sequence for the analysis of the image based and sequence based analysis – we reduced the variation and reading errors for the IBA and the SBA analysis to a possible minimum. One variation left is the possibility that the differences seen in IMT measures between one base image and a sequence of images might be due to motion during the acquisition of the carotid artery images. The ultrasound observer tried very hard to get a stable scan sequence during the 3 seconds of data recording. Due to our ultrasound procedure we think that this possibility is very small.

The remaining difference can be assumed as true difference of both methods.

In this study we found a significant difference between IBA and SBA. The results of Bland-Altman analysis showed that both methods did not detect significantly the same values of IM layers. The image based analysis (IBA) measures a significantly lower IMT value than the sequence based analysis. What is the reason for the difference?

A detection error can be excluded, since we used an automatic system. The obvious and logical answer is that image based analysis did not measure the maximal value of an Intima Media layer during a heart cycle (Finding 1, 2).

However, in epidemiology, it is not the absolute value that is important, but how it ranks subjects as having high or low risk. For example, the CLAS study had an IMT value of 0.60 mm whereas this was a very high risk group. The ARIC study had mean levels of 0.70 mm in middle aged subjects with lower cardiovascular risk. However, the ability to rank subjects using the method is also very relevant. The differences between the results of both studies might be explained by the different detection methods and by the end diastolic time variations.

The CLAS and ARIC studies showed the possibility of IMT measurement as a parameter for to evaluate cardio- and cerebrovascular risk, but they also showed the lack of the actual IMT measurement protocols.

Is a person with a mean IMT of 0.6 mm a person with a higher risk for cardio- or cerebrovascular disease or not? To our knowledge there is no data available which can answer this question. All major studies differ significantly with regard to the baseline values of their subjects. The main reason for this difference might be the measurement method. The answer cannot be solved by the current protocols for image based analysis due to the large number of variations and end diastolic variation errors.

Using a large number of subjects, as was recently done in major IMT studies, can decrease the variation and end diastolic variation errors of other influences on the measurement. Due to the large number of subjects, the statements of the major studies are correct that IMT is a proven parameter of atherosclerotic processes in arteries.

We added a correlation analysis to our study to evaluate the correlation of IBA and SBA to the Framingham CHD risk level and other proven risk parameters. We found a weak to moderate correlation for IBA and a strong association for the SBA for the risk factors. Figure 3 shows that an increase in IMT is better correlated with a higher Framingham risk level for SBA than for IBA. When looking at all data together, there is a high indication that IBA analysis might underestimate the true relations for cardio- and cerebrovascular risk.

However, in this study, we hoped to show that IBA might underestimate the true relations. This statement has also been shown in histopathological studies [29-31], but the actual situation of image based analysis – manual or automatic-might need to be reconsidered (Finding 3).

In conclusion, this study shows that sequential analysis has a higher correlation to risk factors than image based analysis. We showed that the image based analysis might underestimate the true relations of cardio- and cerebrovascular risk.

We suggest using sequential analysis as a method for a robust measurement of IMT and cardiovascular risk.

## Authors' contributions

MS carried out the ultrasound examinations and drafted the manuscript, JH drafted the manuscript, JS developed the computer algorithm, DS carried out ultrasound examinations, AN read the manuscript, HB developed the algorithm and AS-T helped with the study design. All authors read and approved the final manuscript.
